# Visual Landing Based on the Human Depth Perception in Limited Visibility and Failure of Avionic Systems

**DOI:** 10.1155/2022/4320101

**Published:** 2022-04-22

**Authors:** Maryam Mobini, Mehdi Sabzehparvar

**Affiliations:** Department of Aerospace Engineering, Amirkabir University of Technology (Tehran Polytechnic), Tehran, Iran

## Abstract

This paper introduces a novel visual landing system applicable to the accurate landing of commercial aircraft utilizing human depth perception algorithms, named a 3D Model Landing System (3DMLS). The 3DMLS uses a simulation environment for visual landing in the failure of navigation aids/avionics, adverse weather conditions, and limited visibility. To simulate the approach path and surrounding area, the 3DMLS implements both the inertial measurement unit (IMU) and the digital elevation model (DEM). While the aircraft is in the instrument landing system (ILS) range, the 3DMLS simulates more details of the environment in addition to implementing the DOF depth perception algorithm to provide a clear visual landing path. This path is displayed on a multifunction display in the cockpit for pilots. As the pilot's eye concentrates mostly on the runway location and touch-down point, “the runway” becomes the center of focus in the environment simulation. To display and evaluate the performance of the 3DMLS and depth perception, a landing auto test is also designed and implemented to guide the aircraft along the runway. The flight path is derived simultaneously by comparison of the current aircraft and the runway position. The Unity and MATLAB software are adopted to model the 3DMLS. The accuracy and the quality of the simulated environment in terms of resolution, the field of view, frame per second, and latency are confirmed based on FSTD's visual requirements. Finally, the saliency map toolbox shows that the depth of field (DOF) implementation increases the pilot's concentration resulting in safe landing guidance.

## 1. Introduction

Today, aeronautical engineers are looking for new methods to overcome the problems associated with limited pilot visibility, by introducing many new measurements and avionics (e.g., aircraft status indicators, radio navigation, inertial landing system, and ground proximity warning systems) [1]. However, landing is still one of the most accident-prone flight phases, with a relatively high percentage of fatal and nonfatal air accidents [1, 2]. It has been reported that almost half of plane crashes occur in the approach and final landing stages [3]. If visibility is limited, the pilot will land using avionics and precision instrument landing systems. However, if these systems are damaged nonexistent or under adverse weather conditions, they will mislead the pilots and lead the pilots to accidents on controlled flight into terrain (CFIT). In commercial air transport alone, more than 30% of fatal accidents worldwide are classified in the CFIT, where the aircraft hits the ground or obstacles because of the lack of external visual reference or knowledge of the ground/danger situation [1].

On the contrary, weather-related aviation accidents are one of the most significant causes for concern in aviation safety [4]. Adverse weather conditions lower than required for visual flight rule operations are the most significant factors influencing airport delays and reduced runway capacity [1, 5]. Low visibility is the leading cause of flight accidents and disorder in flight planning [6]. The pilot behavior in the face of adverse weather is an old problem [4]. Pilots' performance and perceived mental workload are a function of expertise and flight conditions [7]. The pilot needs help to land safely under any adverse weather conditions. Pilots rely on external visual cues during the landing phase [7]. In a report from the flight crew, cited in [8], the pilots stated that, at the time of landing, after encountering glare, flash, etc., they were unable to judge the distances (depth perception). Also, some pilots had doubts about using avionics or seeing the lights on the runway due to fog, dust, heavy rain, or freezing temperature. According to the pilots' statements, Tušl et al. [9] provided suggestions for improvements of external visual cues; this shows the shortcomings of current avionics in the perception of low altitude, approach, and landing distances. The new suggestions for a visual landing system design are necessary because there is not sufficient natural vision for the flight crew [10].

Vision-based landing navigation and guidance, which have the benefits of accuracy, independence, and low cost, have been favored in air transport for many decades; many developments have taken place in sensor technology, processing hardware, and air transport requirements [11, 12]. All methods show significant improvements in vision-based landing guidance, but dealing with methods of the depth perception of the human eye can be effective in a safe landing. More similarity of the virtual images to the pilot's eyes will provide a timely and appropriate decision, which requires better understanding and then implementing human vision cues on virtual images.

Addressing the human vision cues and depth perception in piloting, driving, and surgery is a concern for researchers. One approach to the depth perception method is to adjust the eye lens, i.e., accommodation. The accommodation of the eye lens shows the state of muscle tension in which the eye lens focuses on the target [13, 14]. The performance of the accommodation or the depth of field (DOF) is similar to the function of the camera; the presence of DOF increases the realism of rendered images and helps to a better depth perception [15].

The primary purpose of this research is to create a 3D model landing system (3DMLS), help the pilot, and have a safe landing on the runway, without any additional equipment installed on the pilot's head. The 3DMLS displays this landing environment (a combination of dynamic and static information). This system could be activated when ILS is out of service. Above ground level (AGL), simulation is based on the airport geographical coordinates and the environment map. As the aircraft height decreases, simulation is based on available data and sensor information. By setting ILS frequency, the 3DMLS is displayed in the cockpit and the DOF cue is implemented on the selected area (the attention position/runway) in simulation.

On the visual attention area in flight, research results in 2020 revealed that the pilot experience level had a significant impact on the fixation time ratio and dwell time [16]. Compared to novices, professional pilots had a higher perceptual efficiency (more numerous and shorter dwells), a better distribution of attention, an ambient mode of visual attention, and more complex and elaborate visual scanning patterns [17]. When the autopilot is off, pilots should allocate visual resources (e.g., out-of-the-window) [16]. Other research results in 2017 show that the pilots did tune on the runway and the runway direction, during approach and landing [18]. Accordingly in the past literature, runway detection methods to find the airport location were used [19, 20], but in this study, the airport and runway are simulated and the depth perception algorithm is applied to display the airport's location. Identification of objects on the runway is conducted by an external sensor installed on the aircraft. Following [11], the IR sensor is used for appropriate performance under various ambient light and adverse weather conditions. Therefore, the pilot's eye is modeled statically and dynamically.

A recent study by Entzinger and Suzuki [21] constructed a model of a human pilot. However, it did not implement the accommodation cue in visual cues. Other studies have combined synthetic vision systems (SVS) and enhanced flight vision systems (EFVS) [3]. Artificial vision data are utilized to identify dynamic objects on the runway surface [22]. A combination of natural and virtual information is utilized to enhance airport scenes. The previous research uses a combination of accurate sensor information and virtual images similar to SVS and improves the pilot's visual information in decision-making. Given that the pilot has a great impact on the approach and his fatigue, skill, and sensitivity affect decision-making [23], the main aim is to look for a system that has a positive effect on making the right decision and reducing pilot workload. Therefore, a landing area has been simulated and displayed in the cockpit.

The novelty of this study is the design of a visual landing system by simulation of the airport and surrounding area, called the 3D model landing system. The 3DMLS uses a human depth perception algorithm to focus on the runway. The 3DMLS will be significant when the ILS is out of service. This method will not take the pilot out of normal mode. It only requires the multifunction display of the aircraft. To display and evaluate the system performance, a landing auto test is also designed. In the landing auto test, the glide slope is derived by comparison of the position of the aircraft and the airport. The aircraft moves according to the flight motion equations on the glide slope. Finally, the quality of the simulation images is shown by the visual standard requirement section of CS-FSTD (A). Also, to evaluate the attention, the MATLAB saliency toolbox [24] is used to assess concentration after applying the depth-of-field (DOF) cue.

The main contributions presented in this study are as follows. In Section 2, the related work on this topic is presented. In Section 3, the depth vision methods are thoroughly discussed, and the landing geometry is described. In Section 4, digital elevation model (DEM) simulation, runway and surrounding simulation with the implementation of a depth perception algorithm, and the design of landing auto test are presented. The evaluation and conclusion are presented in Section 5.

## 2. Related Work

### 2.1. Visual Navigation Instruments in Landing

In current flights, navigation instruments and warning systems on the flight path (Ground Proximity Warning System (GPWS) and Terrain Avoidance and Warning System (TAWS)) and, at lower altitudes, ILS, along with the pilot's natural vision, provide accurate information about the position, attitude, and flight environment. Recent research has shown that a pilot's inadequate visual perception can endanger the landing phase [2]. The lack of advanced avionics on the runway, due to the infrastructure [11], and failure to provide accurate information by sensors, as well as a lack of proper runway visibility under adverse weather conditions, can lead to fatal errors in distance perception between obstacles and terrain in visual landing.

In the field of visual landing, Chen et al. proposed a rapid autonomous landing strategy in GNSS-denied environments using the visual system. Through the status of the airborne camera and image information, the relative position information between the UAV and landing point can be obtained for navigation [25]. In 2021, Safe Landing Zones (SLZ) in crowded scenarios were proposed. To do so, the occupancy map was projected, and the study showed how to prevent the UAV from hurting people during an emergency landing [26]. In the field of using the human vision method in the landing phase, a precise landing algorithm for UAVs based on an improved binocular visual SLAM system was proposed. The direct linear method was used to solve the pose between 3D and 2D images, and the accuracy and effectiveness of the method were further verified by the simulation results in the actual dataset [27]. Mazenc et al. [28] proposed vision-based guidance, navigation, and control solutions to increase flight safety during near-ground operations in commercial aircraft other than UAVs. For commercial aircraft, vision-based landing is divided into two categories: ground-based and onboard-based [29, 30]. On the contrary, onboard-based vision landing navigation based on forward-looking images and computer vision algorithms can be divided into two types, namely, moving platform-based [31] and airport runway-based methods [32]. In this study, onboard-based visual landing for commercial aircraft is examined. Because the test method for human-crewed aircraft is hazardous and costly, mostly simulations using preflight tests are used to test such techniques during the approach and landing phases.

Although the literature shows significant advances in vision-based landing navigation, there are still significant problems in perceiving the landing environment that need to be coped with [11]. Finally, considering the importance of human vision quality, details must be considered, especially depth perception. In the following, the methods of depth perception are discussed so that one of them can be selected and implemented.

### 2.2. Deep Perception

Understanding visual cues is a way to understand depth perception and natural human vision. In general, we have three categories for depth perception cues. It is essential to address each of the categories in a specific application [13]. In [14], two categories of physiological and psychological depth cues are mentioned. While the psychological depth cues are used to perceive two-dimensional objects, the physiological cues are only used for objects that are genuinely three dimensional [14]. The psychological depth cues include linear perspective, shading, texture, and prior knowledge [33]. Four physical depth perception cues are used by the human brain: convergence, motion parallax, binocular disparity (stereo), and accommodation. In this study, among the depth perception cues, accommodation or depth of field is considered. The accommodation is similar to the lens function. Although the use of glasses with a blur filter has not been appropriate [34], we claim that blurring the surroundings can help the depth perception and concentration on a selected area.

## 3. Methods

### 3.1. Overview of the Method

**Figure 1:**
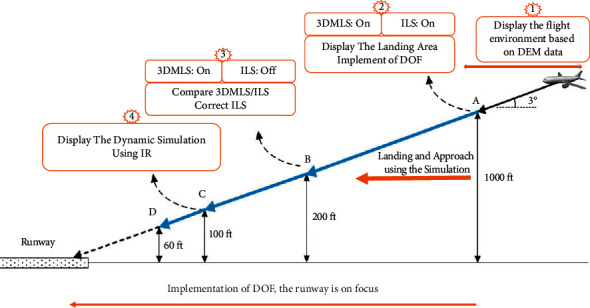
Simulation steps of visual landing based on the human depth perception. In step 1, the suggested system (3DMLS) displays the environment without the details. In step 2, 3DMLS and the instrument landing system are on. The 3DMLS simulates more details of the environment in addition to implementing the DOF algorithm. In step 3, ILS is off. In step 4, infrared data are used to simulate and are displayed on the multidisplay monitor.

The 3D model landing system is used under limited visibility, failure of navigation aids/avionics, or adverse weather conditions. In 3DMLS, the landing area is simulated based on the digital elevation model and INS data. According to INS data, DEM data are selected and simulated. This simulation focuses on the runway utilizing the human depth perception algorithm, DOF. The simulation focusing on the runway is displayed on the multifunction sisplay system.  Figure 1 shows the simulation steps.

**Figure 2:**
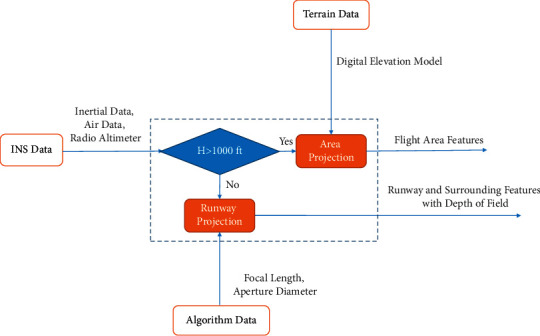
The frame of the proposed landing system. The DOF cue implements at an altitude below 300 m AGL. The blue-dash line shows the human vision model and simulates flight path, runways, and surrounding area.

When the approach begins, the 3D model landing system will be simulated based on the camera's position (user). First, the environment simulation without the detail is displayed. As the aircraft height decreases, the landing area is simulated and the DOF cue is implemented.  Figure 2 shows the block diagram of 3DMLS. The system outputs are flight area features, and finally, runway features with DOF are called the human vision model. The human vision model is used for depth perception and human visual attention investigation.

**Figure 3:**
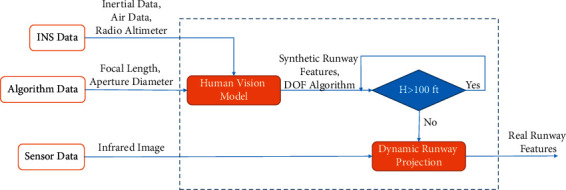
The block diagram of runway projection. The blue-dash line is building the landing area and real runway projection. The inputs are INS data, algorithm data, and sensor data to simulate the dynamic runway.

Figure 2 shows that the output of the human vision model is a synthetic runway with the DOF algorithm. The human vision model combines IMU, BARO, and RALT, a processing unit, and the depth of field parameters. As the aircraft height decreases, using an infrared image, the dynamic environment is simulated.  Figure 3 shows the block diagram of a real runway simulation. The inputs are INS data, DOF algorithm parameters, and sensor data.

**Figure 4:**
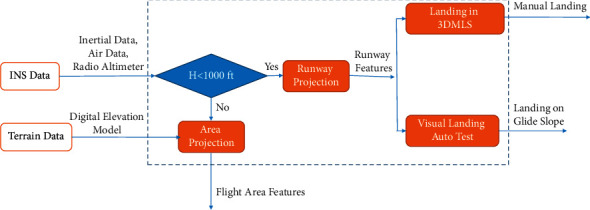
Block diagram of manual landing and landing auto test. Landing on the glide slope (landing auto test) is designed to analyze the system.

To display system performance and have an accurate and steady landing path, the aircraft moves according to the flight motion equations on the glideslope, called the landing auto test. The user can choose the ILS or landing auto test. The landing auto test is similar to the ILS, but the height is determined according to the simulation. As the altitude decreases, the landing auto test will control the aircraft toward the touch-down point on the steady path.  Figure 4 shows the height at which the landing auto test is designed. The outputs are the manual and auto test landings in the flight area simulation.

Since the novelty of this study is the simulation of the airport and surrounding area for visual landing, a landing environment has been simulated. This simulation uses the human depth perception algorithm to reduce pilot workload, model the human eye, and focus on the runway during landing. Finally, FLIR camera data are used to simulate the runway and vehicles around it, where the calculation of the FLIR camera has been ignored. There are four main steps in implementing this idea: simulation of the airfield area by using DEM data (Section 4.1), the runway simulation and the surrounding items with the DOF (Section 4.2), dynamic runway simulation (Section 4.3), and design of the landing auto test to evaluate the simulation performance (Section 4.4). The implementation of DOF and the geometry of landing are described in the sections below.

### 3.2. Depth of Field

There are two main groups in the implementation of DOF in 3D rendering applications: object-space and image-space. The first group works in object-space and calculates DOF directly. This can be done using accurate camera models that mimic the aperture and focal length. This method creates an actual image and produces accurate results. The object-space method is applied to full-focus images, and these images are blurred by the depth map. The depth map is used in conjunction with the camera model to determine the blurring of each pixel. In general, object-space approaches produce more realistic results than image-space approaches and do not have artifacts. The object-space technique is costly and not suitable for real-time rendering [35]. The second category uses postprocessing methods. The image is first rendered with complete focus, and the depth map is used to calculate the amount of blur to be applied to each pixel. Image-space approaches are much faster than object-space approaches, but they will not work well in all situations [35]. Because speed is vital in our intended application and a depth map of all situations is available, image space approaches are selected.

**Figure 5:**
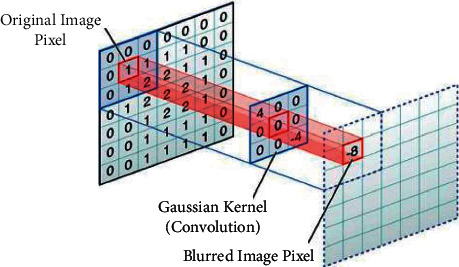
Convolution operation on a 7 × 7 matrix with a 3 × 3 kernel. The output is blurred image pixels.

The depth of field in the postprocessing method is suitable for interactive applications such as virtual reality. Rokita introduced a method to convolving the image with the 3 × 3 kernel and created a blurred image. Although this method is faster than direct filtering, PSF is limited to the Gaussian kernel [35]. In this way, each point convolves with a Gaussian kernel and is added together (Figure 5). The two-dimensional Gaussian kernel is written as follows, and the central pixel has the most weight:

(1) $$\begin{array}{c} \text{Gaussian}\_\text{Filter}=\frac{1}{2\pi \sigma^{2}}e^{-\left(x^{2}+y^{2}\right)/2\sigma^{2}}. \end{array}$$

Assume the kernel is 3*×*3 and Sigma is equal to 0.3. Gaussian Kernel is

(2) $$\begin{array}{c} \text{Gaussian}\_\text{Kernel}=\frac{1}{2\pi \sigma^{2}}=\frac{1}{2\times 3.14\times 0.6\times 0.6}=\frac{1}{2.2619},\\ X=3⟶X=\left[\begin{array}{ccc} -1 & 0 & 1\\ -1 & 0 & 1\\ -1 & 0 & 1 \end{array}\right],\\ Y=3⟶Y==\left[\begin{array}{ccc} -1 & -1 & -1\\ 0 & 0 & 0\\ 1 & 1 & 1 \end{array}\right],\\ \frac{-\left(x^{2}+y^{2}\right)}{2\sigma^{2}}=\left[\begin{array}{ccc} -2.77 & -1.38 & -2.77\\ -1.380 & 0 & -1.38\\ -2.77 & -1.38 & -2.77 \end{array}\right]\Rightarrow \text{Gaussian}\_\text{Kernel}=\left[\begin{array}{ccc} 0.027 & 0.11 & 0.027\\ 0.11 & 0.44 & 0.11\\ 0.027 & 0.11 & 0.027 \end{array}\right]. \end{array}$$

In this study, according to Hillaire's idea in the automatic adjustment of the depth of vision, a rectangular area is considered for points in focus. The Gaussian filter determines each pixel's weight. More weight is allocated to critical objects.

### 3.3. The Geometry of the Landing

**Figure 6:**
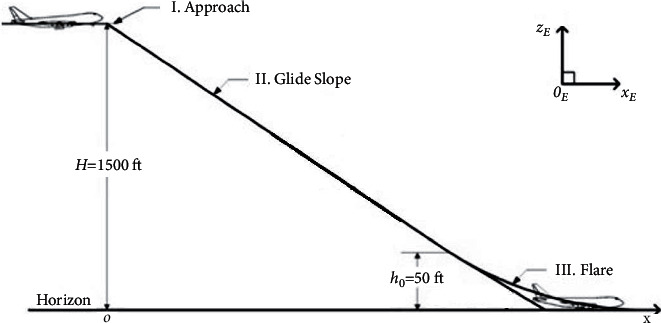
Landing slope; three landing phases: initial approach, glide slope, and flare.

A typical landing includes three phases: initial approach, glide slope, and flare. According to Figure 6, the pilot descends from the cruise altitude to approximately 420 m (∼1500 ft) AGL for heavy aircraft or less than 420 m AGL for light aircraft (the approach phase). The pilot then positions the aircraft so that it is heading towards the runway centerline in the glideslope phase. As the aircraft descends along the glide slope path, its pitch, attitude, and speed must be controlled. The descent rate is about 3 m/s, and the pitch angle is between −5 to 5 degrees. As the aircraft descends to 7–30 m above the ground (the maximum value for a Boeing 747), the slope angle control system disengages, and a flare maneuver was executed between 0 and 5 degrees for most aircraft [36]. Among the three stages of landing, the glide slope is the most demanding one, which is the focus of this study, and the flare phase is omitted.

Since the main obstacle in the design of complex motion is the lack of the necessary mathematical apparatus [37] and the main purpose of the manuscript is to create 3DMLS landing safely on the runway, it is not necessary to model nonlinear coupled equations in the landing phase. Simple and linear equations in the stability frame can well express the motion in the landing phase, and the rest of the equations are omitted.

The commanded (calculated) altitude *H*_*c*_ and the real altitude *H*, respectively, are

(3) $$\begin{array}{c} {\dot{H}}_{c}=V_{0}\sin\;\gamma_{c}\approx V_{0}\gamma_{c},\\ \dot{H}=V_{0}\sin\;\gamma \approx V_{0}\gamma =V_{0}\left(\theta -\alpha \right), \end{array}$$

where *γ*=(*θ* − *α*), the natural slope angle of the aircraft trajectory is during landing, *γ*_*c*_=(*θ* − *α*) is the commanded value of this angle, *V*_0_ is the nominal flight speed, *α* is the aircraft attack angle, and *θ* is the aircraft pitch angle; because the slope angle, expressed in radians, has small values, the approximations sin  *γ*_*c*_ ≈ *γ*_*c*_ and sin  *γ* ≈ *γ* have been used.

**Figure 7:**
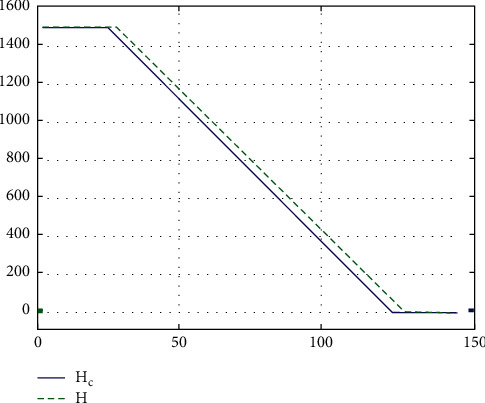
The simulation of the second phase (the glide slope) in MATLAB.

The values of the parameters are according to [36] for the Boeing 747. This section simulation in MATLAB/Simulink software is shown in Figure 7.

According to the above equations and Figure 7, the camera moves in the simulated environment.

## 4. Simulation

### 4.1. Simulation of the Flight Area

**Figure 8:**
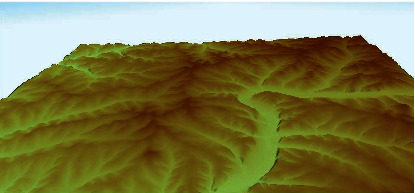
Simulation of DEM data before the approach phase.

As shown in Figure 1, at altitudes above 300 m AGL (less than 420 m AGL), when the aircraft has not yet entered the approach phase, the pilot's vision is implemented based on the altitude information of the environment. The environment is simulated using the data at https://www.webGIS.com.  Figure 8 shows the simulation of this section in MATLAB/Simulink. The information of the gauges, barometer, and radio altimeter is used for the flight area simulation.

### 4.2. The Simulation of the Runway and the Surrounding

**Figure 9:**
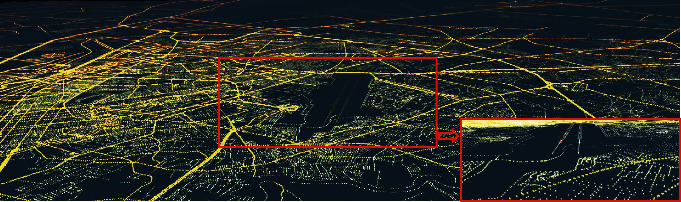
Simulation of flight field with the depth perception algorithm. The algorithm is executed on the selected part of the image. The red box shows the lights in more detail.

Unity software is used to display approach details. In this environment, according to Figure 2, at fewer than 300 m AGL, DOF has been implemented. The Gaussian filter is selected and implemented as a postrendered DOF cue.  Figure 9 shows the approach area focusing on the runway and simulates the runway light. The camera's movement is in line with the movement of the aircraft.

Since the view is vast, the DOF cue shows that the viewer is focused on the runway. Despite the distortion from the direct landing path, the runway is in focus. It is possible to remove or reduce the distortion manually.

### 4.3. Dynamic Runway Simulation

**Figure 10:**
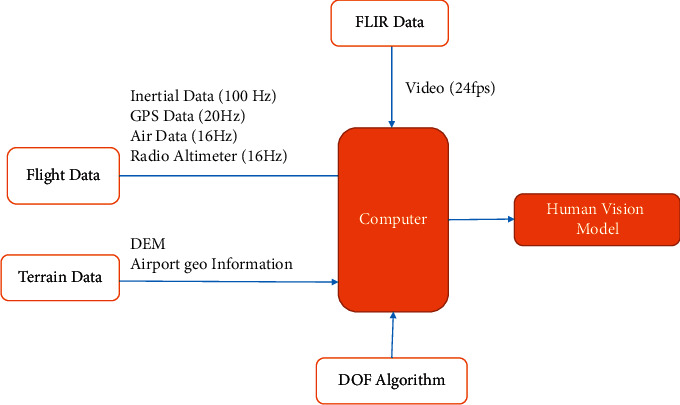
Block diagram of the depth perception algorithm implementation with the FLIR camera. The inputs are flight data, terrain data, camera data, and the DOF algorithm.

Landing is often done according to the pilot's vision at an altitude of fewer than 30 m AGL. Peinecke and Schmerwitz [38] detail the implement of a real-time enabled simulation of the infrared image for landing and concentrate especially under fog conditions. In this study, the forward-looking infrared camera, with a rate of 24 frames per second, is used at a low altitude. This study uses the camera data in [11]. In this reference, the position and attitude error of measuring is less than other methods (less than 1 meter per axis for altitudes below 30 m AGL). An object on the runway at an altitude of less than 30 m AGL will be visible with this method. The block diagram of the depth perception algorithm implementation is shown in Figure 10.

### 4.4. Design of the Landing Auto Test

**Figure 11:**
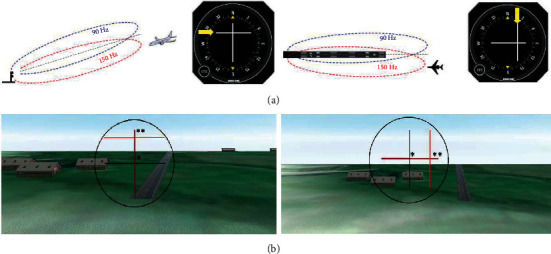
Right: the aircraft is on the left side of the landing path. Left: the aircraft is below the landing path. (a) ILS and (b) 3DMLS. (^*∗*^) indicates the aircraft location (the intersection of two black lines) and (^*∗∗*^) indicates the location where the aircraft must be positioned to land properly (the intersection of two red lines).

The proposed 3DMLS is an alternative system to existing systems. To display, evaluate, and check the performance of the 3DMLS and depth perception under the same conditions, a landing auto test is designed and implemented. The landing auto test creates an accurate and steady landing path. The landing auto test guides the aircraft along the runway based on the solution of the equations of motion on the glideslope. The glide path is derived simultaneously by comparison of the current aircraft and the runway position (because a 3D runway model is available [19]). Those positions are available based on image data. The proposed system is similar to ILS and is displayed on the multidisplay monitor shown in Figure 11.

Figure 11 shows the operation of the ILS and landing auto test under the same conditions.

## 5. Validation and Conclusion

Although ILS is a precision runway approach aid, this system also has problems. Height is an important technical parameter for flight safety in the landing phase [39]. The flight altitude from GPS is usually inaccurate and unreliable, and the height channel of INS tends to diverge, caused by the absence of damping. The air pressure height or radio altitude is adopted to damp the height channel of the INS, but its accuracy is too low to meet the precision landing requirement. This height enters the ILS calculations and causes an error. If the image is more accurate and closer to reality, then the height decision is more accurate and reliable. The landing auto test is employed to create an accurate and steady landing path. The images are captured from the auto test and analyzed. The following are two methods of evaluating the proposed system based on image quality and user attention. However, there are ways to increase the image quality and improve the contrast image using fuzzy logic ([40–42]). In this study, the results are evaluated without changing the image quality.

### 5.1. Validation of Results

Evaluation of results is performed using the standard flight simulator and saliency toolbox 2.3. In the flight simulator, viewing angle, resolution, and number of frames per second are examined.

#### 5.1.1. Flight Simulator Standard

**Figure 12:**
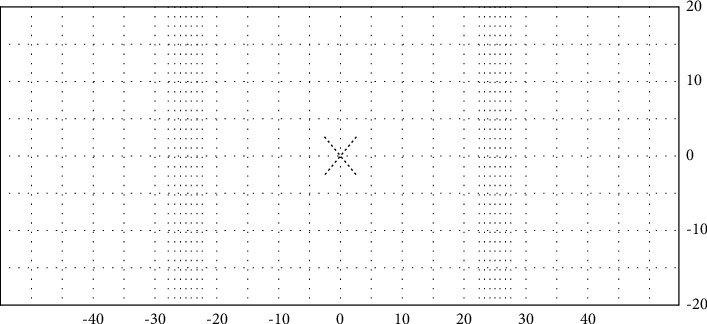
Example of a spherical grid test pattern [43].

**Figure 13:**
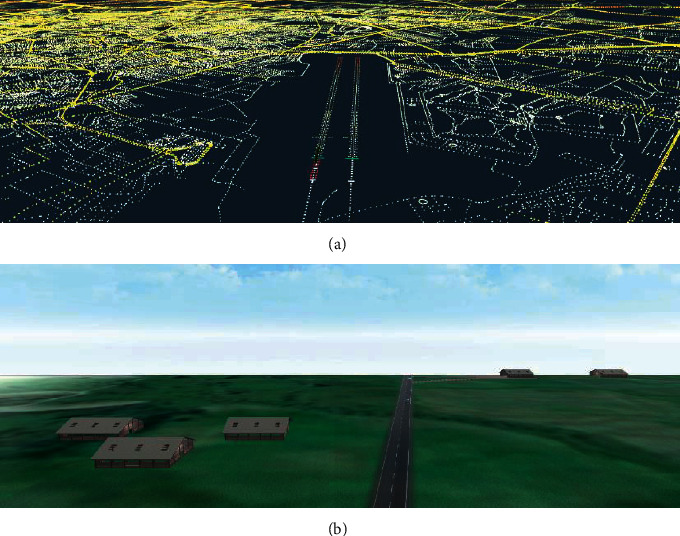
(a) Simulation of lights at night and (b) simulation of the runway, 88 degrees horizontal field of view on either side of the center of the design eye point and a 36-degrees vertical field of view. The minimum collimated visual field of view for each pilot is 180 degrees horizontal and 40 degrees vertical.

In this section, items such as field of view angle, number of frames per second, and resolution in the simulation are evaluated:

- The visual field of view: in a flight simulator, the verification that the visual system field of view is adequate can be checked by the use of a theodolite. The setup of such equipment can be elaborate during the initial system test. A typical procedure for this test is to provide a grid pattern of lines or light points that subtend 5 degrees per line/point allowing the number of squares to be counted to demonstrate the required field of view. The squares should appear square, not rectangular. If there is doubt about the angle subtended, a theodolite should be used to prove the exact field of view. The adequacy of the system is determined by the evaluator (s) performing the test [43]. An example of a spherical grid test pattern is shown in Figure 12.
- On the contrary, in the simulated environment, the field of view is 360 degrees. The camera displays approximately 70 degrees horizontally and 30 degrees vertically; this angle is expandable. The FLIR camera also has a limited field of view (20° horizontally and 30° vertically). According to CS-FSTD (A) [44] (continuous, cross-cockpit, and minimum collimated visual field of view providing each pilot with 180 degrees horizontal and 40 degrees vertical field of view for Class C & D), this constraint cannot be a problem because this camera is used to complete the runway information at low altitudes.  Figure 13 shows the runway with 88 degrees horizontal and 36 degrees vertical field of view.
- Frames per second: the frame rate in the flight simulator is 150 milliseconds. The camera's shooting rate per second in the simulated environment is 24 frames per second, so this simulation satisfies FSTD Certification.
- Resolution: the resolution is expressed with pixel density, but the perceived quality depends on the pixel density and the viewing distance. The closer we get to the screen, the more clearly our eyes will distinguish its respective pixels. This resolution is called angular resolution. According to the equation, reducing the angle *S* (arc min) improves the perceived quality, and this can be achieved by increasing the pixel density, decreasing *s* (the pixel size (the pixel size is the opposite of the pixel density)), and increasing the distance (*d*). Normal vision is “20/20” and can see a maximum of 5 arc mins (1 arc min equals 1/60 degree):

(4) $$\begin{array}{c} S=2\;\tan^{-1}\left(\frac{s}{2\;d}\right). \end{array}$$

**Figure 14:**
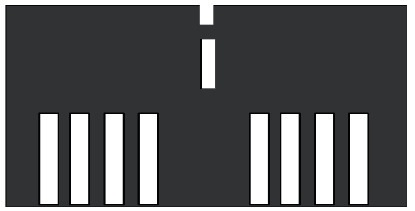
Example of a surface resolution test pattern [43].

It should be noted that the resolution check is one of the display tests in the flight simulator. In the simulator, the method of verification that the surface resolution of a visual system is adequate should not be solely by a method that relies on the use of the human eye. The eye should be positioned on a 3-degree glide slope∼2000 m slant range from the centrally located threshold of a black runway surface. An example of a surface resolution test pattern is shown in Figure 14. The test pattern consists of a black runway surface 300 m long and 6 m wide, the origin of which is located at the center of the runway. The white threshold bars are 4.8 m wide with 1.2 m gaps in between. At this range, the gaps subtend two arc minutes to the eyepoint [43].

Based on the FSTD certification standard, the maximum angular resolution is 2 arc min. Assuming the pixel resolution is 1366*∗*768 for the 13-inch monitor and the distance to the monitor is 0.3 m (11.81 inches), we will have a pixel size of 0.00828 and an angle resolution of 0.04. Thus, the hardware and software satisfy the FSTD Certification.

#### 5.1.2. Saliency Map

The pilots' focus on the airport and the runway as they approach; addressing the methods of eye depth perception will increase the pilots' attention on the landing area and help them make timely and appropriate decisions. The user's visual attention should be measured after applying the cue. One of the practical methods to evaluate the pilots' attention is an eye tracker. At the beginning of this study, the results were used to detect the pilots' attention during landing and approach. The second method is software evaluation. Bottom-up visual attention models have been used to evaluate the proposed method. One of the best models is the saliency toolbox 3.2. The attention quality on the runway is shown and analyzed by the use of the saliency map toolbox in MATLAB. Using the landing auto test, the image was saved in the specified position exactly. Both images (applying the algorithm to the camera and the camera without applying a filter) were given as input to the toolbox, and the following results were obtained.

**Figure 15:**
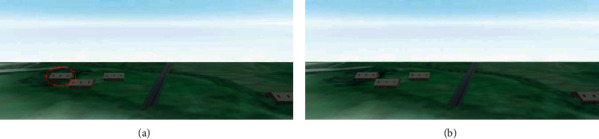
(a) Image without DOF; (b) image with DOF. The target, shown with the red circle in the left image, is the first attention in the right image.

As shown in Figure 15, the selected target, shown with the red circle, is the first focus option in the right image. According to the saliency map, the initial options, the surrounding buildings of the runway, are not the user's attention, but by applying the proposed algorithm, the pilot turns his attention to the runway and the surrounding space. By running the model several times, it is observed that the field of view and the position of the camera affect the user's attention. As well as DOF parameters such as aperture diameter and focal length affect the user's attention. The target object's saliency map will be more significant after applying DOF than before applying the cue. Assigning a more significant number to objects indicates more viewer attention. Therefore, the saliency map also shows that DOF implementation increases the user concentration for safe landing guidance.

### 5.2. Conclusions, Constraints, and Future Works

This study proposes a new visual landing system called the 3D model landing system (3DMLS), developed based on a depth perception algorithm, i.e., depth of field. The 3DMLS is the simulation environment for visual landing and is displayed in the cockpit. This system can perform under adverse weather conditions, limited visibility, ILS failure, or lack of instrument landing system. In the ILS range, the 3DMLS simulates more details of the environment in addition to implementing the DOF algorithm. To display and evaluate the performance of the 3DMLS and depth perception, a landing auto test is designed and implemented. The landing auto test creates an accurate and steady landing path to check the images under the same conditions and verify the results. In the landing auto test, the glide slope is derived by the aircraft's and the airport's simulated position. The method is simulated in the MATLAB/Simulink and unity software environments. Despite methods such as fuzzy logic to enhance the quality and the image contrast, the original images are evaluated. The quality of the simulation images is shown and analyzed by the requirements of the FSTD's visual section. As shown in Table 1, the results confirm the accuracy of the proposed method.

On the contrary, the attention quality on the runway is analyzed and shown by the use of the saliency map toolbox in MATLAB. The focus points in the simulation are the first to fourth choices in the saliency map. The angle of view, distance to the target, the camera options, and DOF adjustment parameters affect the user's attention. By tuning the camera parameters, the selected points (airport or runway) will be more attention. The results show that applying DOF, the saliency map number of each object (which represents the amount of attention) increases. We will have a 5 to 10% increase in the saliency number.

In the simulation, the main factors that affect the accuracy of aircraft motion estimation include sensor calibration error and terrain database precision. Accurate results are also obtainable by adopting a high-precision terrain database, high-quality sensor, and precision glide slope data in the practical test.

Since this study applies to commercial airliner aircraft, the human and financial expenses for flight testing have dropped out. Thus, validation aspects have been authenticated by flight simulator compliance approaches.

Another problem in practical testing for piloted aircraft is the unavailability of the pilot and the lack of their judgment. In the future, some research and practical work can be done to upgrade the system. (1) The method is applied to current simulators that have all flight phases (such as Flight Gear and Microsoft Flight Simulator). (2) Use other methods to implement the depth of field and compare the methods to get the most constructive conclusion in terms of the FSTD standard and visual consideration, as well as a stronger perception of depth. (3) This concept can be applied to train pilots; it also adds overhead displays to help pilots land safely under a variety of weather conditions.

## Figures and Tables

**Table 1:** Evaluate the results.

Parameter	Flight simulator	Suggested method	Simulator description
	Vertical/horizontal	Vertical/horizontal	
The visual field of view	30/45	40/180	To be not less than a total of 176 measured degrees horizontal and not less than a total of 36 measured degrees vertical field of view from the pilot's and copilot's eye points
Frames per second	6.6	24	150 ms or less after controller movement
Resolution	2>	0.04	According to arc min

## Data Availability

No data were used to support this study.
